# Wellbeing and the Lived Experience of Injured Workers Following Finalisation of a Workers’ Compensation Claim

**DOI:** 10.1007/s10926-024-10264-1

**Published:** 2025-01-04

**Authors:** James Weir, Robyn Fary, Samantha Lee, Tim Mitchell, Venerina Johnston, Mary Wyatt, Robert Guthrie, Bronwyn Myers, Darren Beales

**Affiliations:** 1 Faculty of Health Sciences, Curtin School of Allied Health, Curtin University, Perth, WA Australia; 2 Faculty of Health Sciences, Curtin enAble Institute and Curtin School of Allied Health, Curtin University, Perth, WA Australia; 3 Department of Allied Health, SingHealth Polyclinics, Singapore, Singapore; 4 Pain Options, 7 Hardy Street, South Perth, WA Australia; 5 Centre for Health Research, University of Southern Queensland, Ipswich, Australia; 6 School of Health and Medical Sciences, University of Southern Queensland, Ipswich, QLD Australia; 7 Monash Centre for Occupational and Environmental Health (MonCOEH), Monash University, Melbourne, VIC Australia; 8 John Curtin Institute of Public Policy, Curtin University, Perth, WA Australia; 9 Mental Health, Alcohol, Substance Use and Tobacco Research Unit, South African Medical Research Council, Tygerberg, South Africa

**Keywords:** Claim, Compensation, Experience, Finalisation, Qualitative, Work

## Abstract

**Purpose:**

Workers’ compensation claims can negatively affect the wellbeing of injured workers. For some, these negative effects continue beyond finalisation of the workers’ compensation claim. It is unclear what factors influence wellbeing following finalisation of a workers’ compensation claim. Therefore, the aim of this study was to explore wellbeing through the lived experience of individuals who have finalised a workers’ compensation claim in the state of Western Australia.

**Methods:**

A qualitative study with individual, in-depth, semi-structured interviews was performed (*n* = 20, 55% female, average claim length 22.9 months, average time since claim end 33.4 months). Claim finalisation modes included full medical recovery, claim settlement with permanent impairment, direct settlement with the insurer and settlement with the insurer facilitated by a lawyer. The interview schedule was informed by a previous scoping review and cross-sectional survey completed by this research team. Qualitative data were analysed utilising a reflexive, interpretative phenomenological analysis approach.

**Results:**

Five superordinate and their associated subordinate themes were identified; (1) The role of support; (2) Stigma and discrimination; (3) A new normal; (4) The importance of information; and (5) Recommended resources.

**Conclusions:**

Injured workers experience a range of outcomes related to their wellbeing and employment following the finalisation of their workers’ compensation claim. Based on their experiences, resources to facilitate a transition and adjustment to life following a claim have been suggested by injured workers, including information regarding seeking employment, seeking welfare support, educational materials regarding future expectations, and individualised support care packages.

**Supplementary Information:**

The online version contains supplementary material available at 10.1007/s10926-024-10264-1.

## Introduction

Most injured workers (more than 80% in Australia) who have a workers’ compensation claim are well supported by the compensation system and return to work without significant delay [1]. For others though, a compensation claim can be a life changing experience. Negative experience can be characterised by uncertainty, worklessness, needless disability, lifestyle change and a range of other negative impacts during the claim [2–4]. A systematic review of qualitative research of 13 studies regarding injured workers’ interactions between injured workers and stakeholders within workers’ compensation claims, synthesised the growing body of literature demonstrating the negative impacts of being involved in a claim [4]. The review identified six themes, all related to the interactions between insurers and injured workers, which included counterproductive actions, system disorganisation, perceived claims manipulation, access to treatment, co-operative relations and psychosocial consequences. The majority of interactions were determined to be negative, with pathogenic effects, suggesting that these interactions may influence the development of, or worsening of existing disease or illness. However, the synthesis did not explore the experiences of injured workers following the finalisation of a claim.

In a recent systematic review, it was reported that negative effects during a claim may persist beyond claim finalisation [5]. These negative effects include unstable work status, increased reinjury risk, ongoing symptoms, increased mortality, depression and an increased likelihood of experiencing chronic health conditions such as arthritis, intestinal or stomach ulcers and back problems. Only two studies using limited qualitative methodology were identified in that review. The authors of one of these studies, Sears et al., explored the perspectives of injured workers following claim finalisation, finding that injured workers in Washington State, USA, with lower income levels and lower levels of health insurance express interest in being engaged in workplace wellness programs [6]. The authors of the second of these two studies, Sears et al., within the same jurisdiction, indicated that injured workers who had finalised compensation claims reported a range of suggestions to improve workers’ compensation claim processes [7]. These suggestions included improving efficiency and access to services, improving support in navigating the scheme, better communication, improving competence of rehabilitation counsellors and increasing levels of respect. These two studies sourced qualitative data via open-ended questions on a survey, however, did not implement detailed qualitative methodology to gain deeper insight into workers’ thoughts and lived experience following claim finalisation [8]. Further, these studies included people with permanent impairments and a high proportion of unionised workers, which the authors of these studies indicate may limit the generalisability of these studies [6, 7]

Transitioning from the regulated framework of a workers’ compensation claim to a less-structured environment outside of the claim is a significant change that is not well understood from the perspective of workers themselves. The Western Australian workers’ compensation scheme is a statutory, risk-based, no fault system with state government oversight. In the Western Australian system, claim finalisation is implemented for the following reasons: (1) an individual is determined to have met criteria for a successful, full medical recovery, provided with a final medical certificate and returned to pre-injury work; (2) an agreement is reached between an individual and an insurer to exit the claim environment, and an appropriate negotiation for ongoing support (if applicable) outside the claim is undertaken; (3) an approved medical specialist indicates that an individual meets maximum medical improvement [9], at a level less than full medical recovery, resulting in a permanent impairment, which may attract a prescribed amount of financial compensation; or (4) a claim is finalised with the assistance of a lawyer.

Therefore, the purpose of this study was to explore the lived experience of individuals who have finalised a workers’ compensation claim in Western Australia, focusing on the period around the exit from the claim and on life thereafter. Increased understanding of workers’ perspectives of this transition might facilitate the development of appropriate resources and services to facilitate positive outcomes for workers.

## Methods

### Design

An interpretative phenomenological analysis study design was implemented to explore participants’ experience of life following finalisation of a workers’ compensation claim in the state of Western Australia. Interpretative phenomenological analysis attempts to explore the way in which interview participants make sense of their own experience [10]. This design allowed the research team to apply their knowledge and professional experience to develop the interview schedule and critically evaluate the data in a reflexive manner. The interview question framework was informed by important elements discovered from a previously completed scoping review [5] and online cross-sectional survey of injured workers in Western Australia who had finalised a workers’ compensation claim [11]. Individual in-depth interviews were conducted to capture the main features that characterise the participants’ subjective perceptions of their experience. Ethics approval for this project was granted through the Curtin University Human Research Ethics Committee (HRE2023-0121). The study is reported in alignment with recommendations from the Consolidated Criteria for Reporting Qualitative Research (COREQ) [12].

### Participants

Eligibility criteria were individuals 18 years of age or older who had finalised a workers’ compensation claim in Western Australia at least 3 months prior to completing the interview. All claim types, inclusive of musculoskeletal and mental health claims, were included to allow for a range of experiences. Participants were recruited from a previous cross-sectional survey undertaken by the research team [11], or via email from the database of a metropolitan private specialist physiotherapy practice in Perth, Western Australia. The practice focuses on management of injured workers and provides treatment and rehabilitation, as well as offering second opinions for injured workers from other medical and allied health clinics in Western Australia.

### Reflexivity Statement

The interviewer JW is a Western Australian born and trained physiotherapist working in musculoskeletal pain clinical practice. JW has been practising clinically for 18 years and has a postgraduate degree in musculoskeletal pain disorders and has undertaken clinical training in open-ended interviewing. JW has never experienced a workplace injury or claim, however, has worked for 2 ½ years in an injury management advisory role for a large workers’ compensation insurer in Western Australia and has a strong interest in the management of work-related pain during claims and following claim finalisation. JW has worked extensively with persistent musculoskeletal pain disorders in the community, which incorporates a proportion of injured workers following claim finalisation, leading to an interest in this population and the potential impacts this may have on the broader communities of these injured workers. The research team included DB: a male specialist musculoskeletal physiotherapist and senior research fellow with both clinical and research experience related to work health and workers compensation, RF: a senior female research physiotherapist with extensive experience in conducting qualitative research and SL: a practicing female physiotherapist with postgraduate training in musculoskeletal pain disorders and experience in conducting, analysing and publishing qualitative research. The research team frequently met through the interview and data analysis processes, to consider insights and biases related to our own experiences with interviewing, research and involvement in the management of musculoskeletal pain and injured workers. Reflexivity was regularly discussed and reflected upon through these processes.

### Procedure

All interview participants provided written and verbal consent prior to undertaking a recorded interview and completed a brief pre-interview survey using Qualtrics® to provide demographic and characteristics data. This included gender, age, education level, body region injured, industry at the time of the interview, claim duration, time since the end of the claim, employment status at the time of the claim and at the time of the interview, the Personal Wellbeing Score (PWS) [13], the Brief Resilience Scale (BRS) [14] and the Kessler 6 Distress Scale (K6) [15]. The PWS is scored from 0 to 12 where higher scores indicate higher levels of wellbeing. It is derived from the Office of National Statistics ONS4 [16]. The questionnaire includes four questions with reasonable psychometric properties and has previously been used to evaluate wellbeing in injured workers following claim finalisation in Western Australia [11]. The BRS evaluates the ability of an individual to bounce back or recover from stress and consists of six questions, scored using a range of 6–30 [14]. The total sum is divided by the number of questions answered. 1.00–2.99 is indicative of low resilience, 3.00–4.30 is interpreted as normal resilience and 4.31–5.0 is interpreted as high resilience. The K6 is a measure of psychological distress, using six questions about an individual’s emotional state and is scored out of 24, where higher scores indicate higher levels of psychological distress.

The interview process was intended to elucidate further details regarding individual experiences following claim finalisation. Interviews were performed by one researcher (JW). The interview schedule was tested on one participant, for review of the interview schedule and interviewer training by three researchers (SL, DB and RF). The interview schedule was deemed to be appropriate and further interviews were then completed. The interviews were recorded, before being transcribed verbatim and the accuracy of the transcription confirmed by JW and SL. Participants were provided their transcript and given the opportunity to provide additional comments. After four interviews were completed, JW and SL familiarised themselves with the data and commenced open coding. JW and SL then reviewed the interview schedule with one modification made based upon an emergent theme in the data. Following the fifth interview, the interview schedule was iterated following consensus agreement from JW, DB, RF and SL, to allow for better understanding specifically of the participants’ perspective on what wellbeing means to them. Emergent themes were also identified following the ninth and eleventh interviews. The interview schedule is available in supplementary file 1.

### Data Analysis

Data analysis was undertaken by two researchers (JW and SL) independently utilising nVIVO® 14 software to listen to and read transcribed interviews to familiarise themselves with the data. The researchers (JW and SL) conferred following five interviews, developed a working analytical framework based on developing themes and further conferred with RF and DB. This method of analysis has been indicated to be useful for large data sets and is a useful method for identifying similarities and differences in qualitative data [17]. The researchers (JW and SL) conferred again following 10 interviews and 15 interviews to assess consistency in the evaluation and interpretation of the data related to the framework, with further consultation with RF and DB undertaken at these time points. The researchers determined that saturation of theme development occurred following 16 interviews. A further four interviews were undertaken with subsequent analysis to confirm that no other information was forthcoming and saturation was achieved. Following analysis of all 20 interviews, a visual board was developed to evaluate relationships between themes. The researchers JW, RF and DB met to translate the visual board to a working matrix of superordinate and subordinate themes, which were later agreed upon by SL. The researchers (JW and SL) then returned to the interview data before finalising the matrix and inputting quotations from the data that matched themes. Finally, three researchers JW, SL and DB conferred on the matrix, determining some subordinate themes to be similar in nature and appropriate for reduction to a final version.

## Results

### Participant Demographics

Twenty injured workers who had finalised a workers’ compensation claim in Western Australia were interviewed. Interviews were conducted between April 2023 and October 2023 and were undertaken either in person (2/20), via telephone (2/20) or via online meeting platforms (16/20). The average length of interview was 43.6 min. Participants’ mean age (standard deviation (SD) was 52 (9.1) years. Eleven participants were female (55%). Mean (range) claim duration was 25 months (6 to 26) and mean (range) time since claim finalisation was 33 months (8 to 75). All participants had sustained a musculoskeletal injury.

The mean wellbeing score for this group, measured by the PWS, was 7 (3.3), representing a mid-range of wellbeing. The mean level of resilience for this group, measured by the BRS, was 3.5 (0.7) which is within the normal resilience range and mean psychological distress level, measured by the K6, was 7 (5.2), which is within a mid-range. Nine of the twenty participants reported that they had experienced more than 1 prior workers’ compensation claim. Participant individual characteristics and group summary statistics are provided in Table 1.

**Table 1:** Group characteristics of interview participants (*n* = 20)

Participant	Gender	Age	Education level	Body region injured	Industry	Length of claim (months)	Time since claim finalisation (months)	Mode of claim finalisation*	Pre-injury employment status	Current employment status at time of interview	Personal wellbeing score	Brief resilience scale	Kessler 6 psychological distress
1	F	44	University Degree	Right Wrist	Other services	21	34	A	Full time	Employed Full-Time New Employer	10	3.33	10
2	M	54	Technical College/Trade	Left Knee	Electricity, gas, water and waste services	21	40	B	Full time	Unemployed	9	3.83	2
3	M	46	Technical College/Trade	Left Wrist	Arts and recreation services	20	37	B	Full time	Unemployed	5	2.83	9
4	F	67	University Degree	Lower Back	Health care and social assistance (private)	33	15	A	Full time	Unemployed	6	3.33	7
5	M	39	Technical College/Trade	Right shoulder and Right Hand	Transport, postal and warehousing	21	29	A	Full time	Employed Full-Time New Employer	4	3	9
6	F	57	High School	Lower Back	Retail trade	20	11	A	Full Time	Employed Part-Time New Employer	11	3.33	0
7	M	49	Technical College/Trade	Right Shoulder	Retail trade	21	59	A	Full Time	Employed Full-Time New Employer	6	4.67	1
8	M	47	University Degree	Lower Back	Retail trade	22	26	A	Full Time	Employed Full-Time New Employer	12	5	3
9	M	47	Technical College/Trade	Upper Spine (Thoracic), Shoulder (Left), Shoulder (Right)	Mining	13	55	C	Casual	Employed Casual New Employer	4	2.83	7
10	F	57	University Degree	Wrist and Hand (Left), Ankle and Foot (Left)	Other services	26	11	C	Full Time	Employed Full-Time Same Employer New Role	8	3.83	4
11	M	30	University Degree	Right shoulder	Accommodation and food services	8	14	B	Full Time	Employed Part-Time New Employer	4	3	9
12	M	62	Technical College/Trade	Neck, Lumbar Spine, Chest, Right and Left Shoulder, Left Hip and Knee, Left and Right Ankle and Foot, Left Upper Limb Nerve, Left and Right Lower Limb Nerves	Other services	32	75	A	Full Time	Casual New Employer	2	3.5	15
13	F	52	University Degree	Lower Spine (Lumbar), Ankle and Foot (Right),Lower Limb Nerve (Right)	Retail trade	24	12	A	Full Time	Full-Time New Employer	9	2.33	3
14	M	59	Did not complete High School or any other education	Lumbar spine	Transport, postal and warehousing	21	30	B	Full Time	Casual New Employer	4	2.67	9
15	F	56	University Degree	Mental Health, Head, Neck, Face, Wrist and Hand (Left)	Education and training (private)	64	43	A	Part Time	Casual New Employer	7	3.33	10
16	F	47	Technical College/Trade	Lower Spine (Lumbar), Pelvis, Abdomen, Hip (Right),Knee (Right),Lower Limb (Right),Ankle and Foot (Right),Lower Limb Nerve (Right)	Health care and social assistance (private)	23	9	D	Part Time	Unemployed	2	4.33	21
17	M	66	University Degree	Knee (Left), Knee (Right)	Other services	23	8	B	Full Time	Full-Time New Employer	11	4	2
18	M	57	University Degree	Left Shoulder, Right Shoulder	Accommodation and food services	33	30	E	Full-Time Self-Employed	Full-Time Self-Employed	9	3.83	2
19	F	53	University Degree	Lower Spine (Lumbar), Lower Limb Nerve (Left), Lower Limb Nerve (Right)	Education and training (private)	27	71	A	Part Time	Casual New Employer	11	3.83	9
20	F	44	University Degree	Lower Spine (Lumbar)	Health care and social assistance (private)	6	60	F	Part Time	Part-Time Same Employer	10	4	3
Summary Scores	55% Female 45% Male	51.65 Mean 9.10 SD 30–67 Range	55% University 35% Technical College/Trade 5% High School 5% Did not complete High School			24.55 Mean 11.83 SD 6 to 26 Range	33.40 Mean 21.38 SD 8–75 Range		80% Full Time 20% Part Time	40% Full Time 15% Part Time 25% Casual 20% Unemployed	7.15 Mean 3.27 SD 2–12 Range	3.54 Mean 0.68 SD 2.67–5 Range	6.75 Mean 5.20 SD 0–15 Range

The Mean length of interview was 43.6 min

*SD* Standard Deviation

*Mode of claim Finalisation: (A) Claim settled with assistance of lawyer; (B) Claim settled directly with insurer; (C) Claim settled with insurer and Permanent Impairment awarded; (D) Claim settled with assistance of lawyer and Permanent Impairment awarded; (E) Claim settled with insurer through mediation with regulator; and (F) Achieved Final Medical Certification

### Themes

**Fig. 1:**
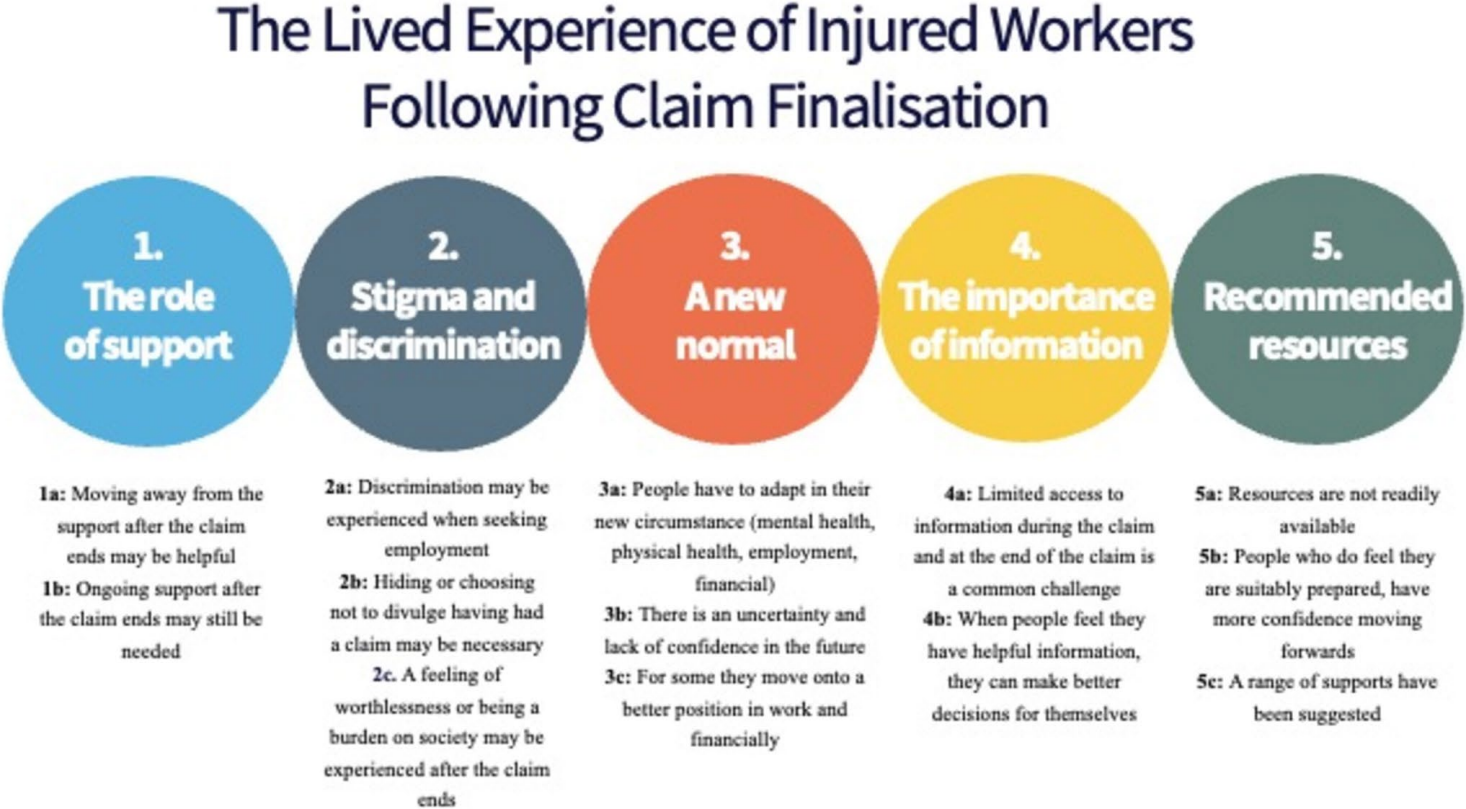
Superordinate and subordinate themes related to the lived experience of injured workers following claim finalisation

Five superordinate and their associated subordinate themes were identified: (1) The role of support; (2) Stigma and discrimination; (3) A new normal; (4) The importance of information; and (5) Recommended resources (Fig. 1). The interview process did elucidate discussion and quotes related to the claim experience for many participants, however, we have intentionally not included this data in the manuscript, in order to focus more on the end point of the claim and experience following the end point of the claim, as per the research question.

### Superordinate Theme 1: The Role of Support

Each participant described their own individual needs for a range of services and support when finalising their claim. In some cases, individual claim finalisation processes and arrangements included ongoing support, including advice from their treating health care professionals, advice from their lawyer, recommendations from vocational rehabilitation providers or advice and support from government-funded authorities and welfare services, however, this was not necessarily the case for all participants. A range of perspectives regarding the role of support following the finalisation of the claim were reported.

### Subordinate Theme 1a: Moving away from the Claim Environment After the Claim Ends may be Helpful

Despite the support that was made available during the claim, participants described the relief and the de-pressurising effect of leaving the claim and the support that was on offer to them, indicating that it was more beneficial to move away from the support that was provided.

“…I got the call to say it's settled and it was such a relief” (P1)

*“It was a huge feeling of relief to not be dealing with that insurance company anymore and the people that I'd had to deal with” (P2)*

Participants reported on the burden they felt of being engaged with the support systems.

*“… not having to negotiate with insurance companies and all their chosen providers, that was good, not having to deal with them any further. That took a weight off.” (P9)*

### Subordinate Theme 1b: Ongoing Support After the Claim Ends may still be Needed

Conversely, participants referred to the need for support that they had received since the finalisation of their claim. Participants expressed a need for ongoing health care and welfare support, due to ongoing disability related to their workplace injury. A shared experience from the participants was of a feeling of abandonment, which was apparent following the claim finalisation. The participants described the challenges they felt related to this, as they attempted to reintegrate into life after the claim.

*“I again was going see my psychologist every two weeks, going to my physiotherapist every two to three weeks, as well to get treatments.” (P1)*

*“They just abandon you.” (P5)*

Some participants reflected on the difficulty they faced whilst trying to access further support.

*“I've got a certain amount of disability, but not enough to get a disability pension, so then I had to go to jobseeker.” (P4)*

*“So for then to suddenly need care. A lot of care. There was nobody, nobody there.” (P16)*

Others indicated the financial challenges and implications of seeking further support following the end of their claim.

*“I'm getting help, the care plan and stuff, from government, which has helped, but it only pays for so much.” (P14)*

### Superordinate Theme 2: Stigma and Discrimination

The participants in this study described their own perceptions of stigma and discrimination that continued beyond the finalisation of their claim.

### Subordinate Theme 2a: Perceived Discrimination may be Experienced when Seeking Employment

Participants reported that despite their desire to find and commence new or alternative employment roles following the finalisation of their claim, they were presented with barriers to do so.

*“I was preparing for interviews and all, … I told them that I got workers’ comp but they did not select me because I did have the workers comp.” (P19)*

### Subordinate Theme 2b: Hiding or Choosing not to Divulge Having had a Claim may be Necessary

In response to these experiences, participants reported that they had intentionally hidden the fact that they had a previous claim, in order to apply for new roles.

*“To be honest, I lied on the application about - Have you ever had a workers’ comp claim? I said no.” (P11)*

### Subordinate Theme 2c: A Feeling of Worthlessness or Being a Burden on Society may be Experienced after the Claim Ends

Participants told of their desire and intention to move forward with their lives following the finalisation of their claim. However, they felt an ongoing sense of discrimination from those around them.

*“I found myself thinking, this, like I've become a burden. So it was like, I really had to dig deep within myself and think, you know, ‘you're a valued person’.” (P13)*

“It really stuffs up with your mind, it really stuffs up. Makes you really worthless.” (P6)

### Superordinate Theme 3: A New Normal

Each participant described their experience of making sense of their circumstance following the finalisation of their claim. For some the process was dynamic and rapid, whilst for others, coming to terms with their ongoing limitations and the required adaptations, is a process that is ongoing.

### Subordinate Theme 3a: People have to Adapt in Their New Circumstance (Mental Health, Physical Health, Employment, Financial)

All of the participants were able to acknowledge that adapting was necessary following the end of their claim. Each experience though, was unique.

*“It's very difficult. I mean a very difficult situation because I have to live with my parents ‘cause I can't afford rent.” (P6)*

“Oh, the biggest challenges have been, obviously, probably mental, personal, financial.” (P14)

Each participant reflected on the nature of their limitations and the impact that those limitations have had on their life since finalising their claim.

*“A lot of my exercise stuff is the gardening and doing activity like … woodwork and that sort of stuff. And no, I can't get back to those so that and the travelling, … I used to go travelling.” (P4)*

### Subordinate Theme 3b: There is an Uncertainty and Lack of Confidence in the Future

Some participants described how along with finalising the claim comes a lack of certainty in what their future holds. It was commonly reported that the transition from a familiar circumstance in life and work to life after the claim was unprecedented.

*“I don't feel very secure…It's life changing. You don't even know what work you can do yet. You don't know how much it's gonna pay you.” (P15)*

“So, to sit there and try and think of another path was, you know, pretty daunting.” (P2)

For some participants who had reported previously having high levels of confidence in their own ability to cope well, this transition was a significant challenge.

*“But the enormous uncertainty of knowing what was going to happen, would I be able to work again, did I have to get knee replacements, when was it going to happen and how was my life going to be affected, that was a huge uncertainty.” (P17)*

### Subordinate Theme 3c: For Some They Move onto a Better Position in Work and Financially

In some circumstances, the participants reported that work and financial situations improved following the finalisation of their claim. A group of participants described retraining or finding a new avenue of employment that was novel and satisfying.

*“It's actually much better, yeah… comparing to what I was doing before and what I was doing after, it was much better.” (P1)*

“I'm on a bigger bracket … I earn more now through my work than I did before.” (P5)

Some participants described that they had transitioned onto an alternative form of compensation, related to income protection support based on their ongoing inability to engage in work.

*“Yeah, well, I pretty much fell on my feet because I've had income protection insurance for a lot of years since” (P2)*

Broadly, across all participants, a level of acceptance was able to be reached, where accommodation and adaptation allowed for progress in life after the finalisation of the claim. The participants described the process in which they had come to accept and live with their limitations, in order to progress and work towards a better future.

*“So those are the kind of things, you know which, when I go out, I should be able to do them, but I can't do them.” (P8)*

### Superordinate Theme 4: The Importance of Information

All participants provided varying accounts about the availability of information pertaining to the post-claim finalisation transition. There was, however, a unanimous agreement on the important role that information has played in their journeys.

### Subordinate Theme 4a: Limited Access to Information During the Claim and at the End of the Claim is a Common Challenge

Aside from the participants who had been engaged in more than one a workers’ compensation claim in the past, each participant described the challenges related to understanding the transition from life within the claim to life following claim finalisation.

*“Oh, probably my lack of knowledge of the whole process. My lack of knowledge of my entitlements. It's like, you know, like a goldfish swimming around in a fish bowl” (P2)*

### Subordinate Theme 4b: When People Feel They have Helpful Information, They can Make Better Decisions for Themselves

It was commonly reported that when information was available, the participants found it easier to make plans and engage in a strategy to move forward.

*“I think the lawyer that I had was pretty good, he, he explained the situation, why they're doing what they do.” (P5)*

*“The only main help I got was from Work Cover WA and they were more than helpful. They returned calls, they gave me some guidance.” (P9)*

The transition from the claim to life after the claim was a significant event for each participant. They were able to articulate the nature of resources they would have found beneficial when reflecting on their experience. These resources, as described by the participants, would differ from the support and services they had previously discovered, in so much as they would be intentionally and specifically related to providing advice, education, support and services related to life following the claim end. However, the majority described a lack of such resources to utilise.

### Subordinate Theme 5a: Resources are Not Readily Available

*“No. Nothing. It was just like, that's it. Sign on the dotted line. Here’s your money. See you later. I was just cut loose.” (P13)*

*“No real information on what I could do if I didn’t want to go down that road. Well, nothing that was clear or that I can remember.” (P3)*

### Subordinate Theme 5b: People who do Feel They Are Suitably Prepared, have More Confidence Moving Forwards

A small proportion of interview participants indicated that they had been able to discover resources that they utilised to their advantage, however, this had required them to be proactive in finding them.

*“You have to be astute at playing the game in dealing with compensation and compensation insurers. Because if you aren’t astute, then you can find yourself heavily disadvantaged, because they will take advantage of you.” (P17)*

### Subordinate Theme 5c: A Range of Supports have been Suggested

The participants were asked *“Do you think educational material would be useful for people finalising a claim. What form should it be in, what type of things should it tell you?”.* All the participants agreed there is no ‘one size fits all’ answer to provision of resources at transition. However, they did suggest a range of resources from brochures to one-on-one support services.

*“I think having almost like a bit of a care package saying, OK, you're transitioning out of Workcover looking after you.” (P16)*

*“You should have a person once you’re settled “this is what's gonna happen, … this is what you can do, what you can claim, [what] you cannot claim” (P5)*

## Discussion

### Summary of Main Findings

The experiences of 20 injured workers who have engaged in and finalised a workers’ compensation claim in Western Australia have been documented. These workers have described a range of experiences and outcomes related to this transition. Each experience and each outcome is individual in nature and varies from positive to negative. The injured workers have shared commentary related to their health outcomes, employment outcomes, stigmatisation and discrimination. The perspectives of the injured workers in this qualitative study suggest that access to information regarding claim finalisation and what to expect following claim finalisation is limited or difficult to access. In some circumstances, moving away from the system provides a positive pathway for moving forwards. In some cases, further support has been needed. The injured workers in this qualitative study have also provided recommendations for further resources, that they consider may facilitate the transition from a workers’ compensation claim to life following finalisation of a claim. These resource recommendations included information regarding seeking employment, information related to seeking welfare support, educational materials regarding future expectations following claim finalisation and individualised support care packages to facilitate the transition.

#### The Lived Experience of Injured Workers Following Workers’ Compensation Claim Finalisation

The injured workers in this qualitative study described a range of perspectives regarding the *role of support* at the time of finalising their claim and support following finalisation of their claim. These perspectives included feeling benefit from moving away from claim support as well as a need for further support. These perspectives reflect recommendations synthesised in a recent scoping review [5] that support accommodation of the individualised nature of workers’ compensation claim experiences. Further, 60% of a group of 335 injured workers with permanent impairments, interviewed regarding their experiences of the workers’ compensation system in Washington State, USA, indicated that increased efficiencies and access to appropriate services were needed [7]. A survey of 374 injured workers in Missouri, USA who, on average, were 72 months since the finalisation of their workers’ compensation claim for low back pain, highlighted long-term pain, catastrophising and pain-related disability for some injured workers [18]. The authors made recommendations to improve the understanding of the individualised nature of the injured workers’ experiences in their claim [18]. These findings suggest that identification and delivery of individualised supports and services may be beneficial for injured workers during and following finalisation of a claim.

*Stigma and discrimination* related to workers’ compensation experiences have been previously described in the literature. The participants in our survey described a perception and experience of feeling obliged to hide their injury and claim history in order to secure employment. This reflects results from a large national survey of 4602 injured workers in Australia, where around one-third of injured workers expected negative repercussions from their colleagues if they disclosed a workplace injury [19, 20]. Approximately fifteen percent of participants in the same survey also reported that their employers actively discouraged reporting of injuries. The negative effects of these experiences of stigma and discrimination in workplace environments may include poorer wellbeing for some injured workers through experiences including stereotyping, as well as detrimental impacts on relationships and mental health [21]. Further to this, in a survey of 1855 ambulance workers, police, fire and rescue workers and emergency service workers in Australia, the potential for poorer recovery from work-related psychological claim injury if stigmatisation was experienced by the injured worker was reported [22]. Therefore, it is likely the presence of a mental health injury alone or in combination with a musculoskeletal injury will result is poorer wellbeing. In a survey of 567 injured workers with permanent impairments, co-worker support, an absence of stigmatisation from colleagues and supervisors, and a supportive workplace environment were associated with reduced return to work interruption and reduced reinjury [23]. These findings suggest that stigma and discrimination towards injured workers can be a detrimental experience during a claim and following finalisation of a claim for some individuals. As there is potential for these experiences to be positively modified through supportive workplace culture and practices, decreasing these practices in the prospective employer population may improve employment seeking experiences for injured workers following claim finalisation.

The injured workers in our study described the nature of a *‘new normal’*. The new normal was different for each individual, but it was commonly characterised by a need to progress towards acceptance. The acceptance process for some injured workers was overwhelming at times, however, it was also perceived as a positive opportunity. Moving away from the claim environment allowed some injured workers to recover a sense of self-efficacy, a valuable element of coping with the new normal. Self-efficacy has been reported as an important characteristic in the prognosis of, and management of outcomes in musculoskeletal pain [24–26]. Contemporary musculoskeletal pain clinical practice guidelines and clinical care standards [27, 28] also encourage education and empowerment of individuals living with musculoskeletal pain, to promote quality of life and health outcomes.

The injured workers in our study related *the importance of information* to their ability to cope with their new normal, yet also reported a limited ability to access information regarding the workers’ compensation system and what to expect after finalisation of their claim. Access to information has also been reported to be a barrier for injured workers during their claim, which when combined with limited understanding of their rights and obligations in the workers’ compensation system, results in injured workers feeling uninformed [29]. Limited access to information and understanding of the complex administrative and technical elements of the compensation system may also contribute to negative influences on locus of control and wellbeing for injured workers [3, 30]. Similar themes relating to the importance of access to education, support and information were also highlighted in qualitative interviews with 18 individuals between the ages of 18 and 65 years old, who had experienced road traffic injuries and were attempting to return to work [31]. Recommendations were made to improve the knowledge of the injured individuals regarding the compensation system, to provide better support to these individuals for returning to work and to improve employer awareness of the importance of return to work. Overall, this suggests that appropriate information, and access to appropriate information, has the potential to promote self-efficacy for injured workers, which may lead to better outcomes following claim finalisation.

The perspectives of the injured workers included *recommended resources*, that they considered would facilitate the transition from a workers’ compensation claim to life following finalisation of a workers’ compensation claim. These resource recommendations reflected the range of individual experiences and points of view, included varied types of resources and formats of resources, as well as varied opinions on who should be considered responsible for providing the resources. Recommendations included the provision of information regarding what to expect following the end of a claim, how to seek employment support, mental health support and financial support. The participants described resources such as brochures, websites or one to one meetings with a professional. The participant group indicated that the responsibility of providing or disseminating the resources might be upon the employer, the insurer or the jurisdictional regulator.

### Strength and Limitations

As far as we are aware, this is the first study to investigate the lived experience of injured workers following finalisation of a workers’ compensation claim. The interview schedule was developed by a research team including academics, practitioners and industry experts with broad research and clinical experience in workers’ compensation, occupational medicine, physical rehabilitation, law and wellbeing. The interview schedule was informed by a previous, robustly designed scoping review [5] and results of a cross-sectional survey [5, 11]. The study has been reported in accordance with the COREQ [32] and adheres to terms and definitions in trustworthy qualitative methodological approaches, specifically; credibility, dependability, transferability, conformability and reflexivity [33]. Gender distribution for the interview participants was balanced and a broad range of industries and musculoskeletal injury regions were represented. Sample size calculation was based upon reaching a point of saturation in the theme development following interview 16, with additional research team consensus to continue to a sample size of 20. The research team considers this to be in keeping with an acceptable qualitative research sample size [34]. As the majority of participants had experienced a musculoskeletal injury, it is possible that the results cannot be generalised to those with mental health injury only or a combination. This study is specific to the experiences of injured workers who have engaged with the Western Australian workers’ compensation scheme and may not be generalisable to other jurisdictions where different claim finalisation mechanisms may exist. The opinions and perceptions of the participants provide valuable insight, however, the potential for recall bias is acknowledged. We have not specifically explored the potential influence of multiple claims on the experience of the injured workers in this interview group. Sixteen of the twenty interview participants had participated in a previous, online cross-sectional survey research project, undertaken by this research team [11], which may have influenced the participants’ reported experiences during the interview process.

### Directions for Further Research

Further understanding is required regarding the lived experience of similar populations in other jurisdictions, including those with musculoskeletal injuries only, mental health claims only and combined musculoskeletal and mental health claims. Further research that explores the experiences of other stakeholders, such as insurers, health care providers and employers, following claim finalisation may also facilitate understanding of this important stage in a compensation claim pathway. Further research may also explore comparisons in experiences between injured workers who have experienced a single claim and injured workers who have experienced multiple claims.

Further research is required to evaluate the nature and quality of the resources that do exist, be they online, in person or in document form, and the nature of accessibility to resources, for injured workers finalising a claim in the Western Australian jurisdiction. If resources are in fact not available, development of consumer informed resources is warranted. Furthermore, if resources are available, yet are difficult to access, consideration should be given to improving accessibility. Industry expert and consumer consensus through a Delphi study approach is one way in which further understanding and direction in this area may be achieved.

## Conclusion

Injured workers in Western Australia experience a range of outcomes related to their wellbeing and employment following the finalisation of their claim. Injured workers have described a range of experiences related to their perceptions of support, stigma and perceived discrimination, their adaptation to life following a claim and their experiences with information related to the transition. Resources that may facilitate a transition to life following a claim have been suggested by injured workers based on their experiences.

## Supplementary Information

Below is the link to the electronic supplementary material.

Supplementary file1 (XLSX 12 KB)

Supplementary file2 (PDF 75 KB)

## Data Availability

The data that support the findings of this study are available from the authors upon reasonable request and with the permission of Curtin University.
