# Supplementary material for: Wellbeing and the Lived Experience of Injured Workers Following Finalisation of a Workers’ Compensation Claim
Source: J Occup Rehabil. 2025 Jan 4;36(1):314–28. doi: 10.1007/s10926-024-10264-1 (PMC12906573; doi:10.1007/s10926-024-10264-1)
Supplement: Supplementary file 2 — Supplementary file2 (PDF 75 KB) [file 10926_2024_10264_MOESM2_ESM.pdf]

The lived experience of people following finalisation of a workers' compensation claim: a qualitative study
Interview Schedule (FINAL)

Introduction:

- Explain the study
- Explain the focus group will be audio recorded and that all participants will be sent a copy of the transcript to check for potential errors. Explain that participants will not be named in the transcript.
- Confirm consent from participants – this will be done via written consent in person or via email

Preamble:

The reason for conducting this interview is to understand your experience as a person who has finalised a workers' compensation claim. We are specifically interested in your views and opinions about:

- Your worker's compensation claim experience
- Your experience of finalising your workers' compensation claim
- Your experience of life after finalising your workers' compensation claim

Before we start, I would like to assure you that all information from this interview is confidential and will only be used to understand more about how people move on with life after finalising a workers' compensation claim. There are no right or wrong answers.

QUESTIONS:

1. What happened during your claim?

- How long did your claim last?
- How long has it been since your claim ended?
- What type of injury did you have?
- Do you blame anyone for your injury?
- How much time off work did you have during your claim?
- What was your experience like during your claim?
    - Did you feel well supported?
    - Did you feel like the people who were involved in your claim dealt with you fairly? If not, were there any in particular who you felt did not treat you fairly?
- What were the biggest challenges for you during you claim?
    - What would have made this easier?

"So now I have some idea of your situation during the claim, I would like to move to the finalisation of your claim."

2. What happened at the time of finalising your claim?
- Were you fully recovered or were you still having problems?
  - If yes to still having problems, were you given any advice?
- How was your claim finalised?
- Did you feel like you were treated fairly?
- What were the challenges for you at the time of finalising your claim?
- Did anyone or anything in particular help you?
- Did anyone or anything in particular hinder you?
- Was there much paperwork? Tell me about it.
- Were you given any advice or educational type material during this time. If so, what was it.
- What would have made this process better for you?
- Do you feel like finalising your claim helped your recovery from your workplace injury?

"Now I have an idea of what happened at the time of finalising the claim, tell me more about your experience since your workers' compensation claim"

3. Tell me about life since finalising your claim
- How did finalising your claim make you feel?
- Have you had to seek treatment for your workers' compensation injury since closing the claim?
- How has finalising your claim affected your work and Income?
- Have you returned to normal exercise since the end of the claim?
- Have you returned to normal social life since the end of the claim?
- How has finalising your claim affected your Relationships?
- What is most different now?
  - What have been the best things about finalising your claim?
  - What have been the most challenging things about finalising your claim?

4. What would have made your journey better?
5. Finally, do you have any advice that you would give to others?
6. What does wellbeing mean to you?
7. Has finalising your claim had a positive or negative impact on your wellbeing?
   - If yes, in what way?
   - If no, in what way?
8. Do you think educational material would be useful for people finalising a claim. What form should it be in, what type of things should it tell you.
